# Vitamin D dynamics predict treatment response to intravenous glucocorticoids in thyroid-associated ophthalmopathy: a retrospective cohort study

**DOI:** 10.3389/fimmu.2026.1778702

**Published:** 2026-04-21

**Authors:** Ling Wang, Jingya Wang, Huayang Xu, Yushi Sun, Xingchen Zhou, Yu Chen, Jiarui Zhao, Jinbo Zhang, Hui Guo, Bingyin Shi, Yue Wang, Meng Zhang

**Affiliations:** 1 Department of Endocrinology, The First Affiliated Hospital of Xi’an Jiaotong University, Xi’an, Shaanxi, China; 2 The First School of Clinical Medicine, Xi'an Jiaotong University, Xi’an, Shaanxi, China; 3 Department of Gastroenterology, Xi’an Jiaotong University Affiliated Children’s Hospital, Xi’an, Shaanxi, China

**Keywords:** biomarker, intravenous glucocorticoids, thyroid-associated ophthalmopathy, treatment outcome, vitamin D deficiency

## Abstract

**Background:**

Intravenous glucocorticoids (IVGC) represent first-line therapy for active moderate-to-severe thyroid-associated ophthalmopathy (TAO). The relationship between vitamin D (VitD) and IVGC efficacy in TAO remains undefined.

**Methods:**

This retrospective study grouped TAO patients completing IVGC therapy with standardized VitD/calcium supplementation at First Affiliated Hospital of Xi’an Jiaotong University (2015–2019). Serum 25-hydroxyvitamin D (25(OH)D) and bone markers were measured pre/post-treatment. Multivariate regression and ROC analysis identified predictors.

**Results:**

In total, 52 TAO patients were enrolled, including 33 responders and 19 non-responders. Non-responders had significantly higher baseline triglycerides, thyroxine and longer duration of ophthalmopathy. Responders exhibited higher baseline β-CTX and N-MID OC. Notably, 73.08% of patients had baseline 25(OH)D deficiency (<20 ng/mL), with a significant 25(OH)D increase observed only in responders (*P* = 0.02). Between-group comparison showed that the treatment-induced change in 25(OH)D (Δ25(OH)D) was significantly greater in responders (*P* = 0.008), who also demonstrated larger improvements in clinical activity score (CAS), NOSPECS score, and diplopia. Multivariate analysis confirmed Δ25(OH)D as an independent predictor after adjustment for confounders (adjusted OR = 1.205, 95% CI:1.033–1.405; *P* = 0.017). ROC analysis showed Δ25(OH)D predicted response (AUC = 0.732, *P* = 0.006), with an optimal cutoff of +2.7 ng/mL. Predictive power was enhanced in males, patients ≥50 years, and baseline 25(OH)D -deficient subgroups.

**Conclusion:**

Δ25(OH)D is an independent predictor of IVGC response in TAO. Monitoring dynamic changes in serum 25(OH)D during therapy and targeting a minimum increase of 2.7 ng/mL may be associated with improved treatment outcomes, particularly in male, elderly, and VitD-deficient subpopulations.

## 1 Introduction

Thyroid-associated ophthalmopathy (TAO), also called Graves’ ophthalmopathy (GO), is the most common extrathyroidal complication of Grave’s disease (GD), and the most common orbital disease of adults. TAO is a potentially sight-threatening disease, which manifested as photophobia, double vision, and corneal ulceration or compressive optic neuropathy in severe disease. Beyond visual impairment, proptosis, upper eyelid retraction, and strabismus caused by TAO frequently lead to permanent facial disfigurement, which significantly impairs patients’ psychosocial health and quality of life (1–3).

Stopping the orbital inflammation and reducing the subsequent tissue remodeling is important for the treatment of TAO (4). Intravenous glucocorticoids (IVGC) were recommended as the first-line treatment for active moderate-to-severe TAO, although new drugs such as teprotumumab, rituximab, tocilizumab, rapamycin, and azathioprine have been shown to be effective (5–8). However, the clinical response rate remained 50%-60% (9). Moreover, systemic IVGC therapy entails a considerable risk of adverse events, which include hyperglycemia, hypertension, osteoporosis, and gastrointestinal mucosal injury (10–12). Early identification of patients sensitive to IVGC therapy is critical to optimizing individualized treatment, improving clinical outcomes, minimizing adverse effects, and reducing unnecessary medical cost. Thus, it is very important to find factors associated with IVGC response, which benefited the exploration of pathogenesis and the management of the TAO. Vitamin D_3_ (cholecalciferol, VitD) has been confirmed to be potent in anti-inflammatory, antioxidant (13), and is recognized as an immune-regulatory hormone (14). Recently, numerous studies have demonstrated the significant link between VitD and GD (15, 16). GD patients exhibited a higher prevalence of VitD deficiency, and 25(OH) D levels were reported to correlate with the thyroid volume (17). The VitD levels were reported to be important in the response to the treatment of antithyroid drugs (ATD), with the lower levels associated with a lower likelihood of remission and a higher relapse rate (18). Furthermore, the VitD deficiency was also an independent risk factor for predicting failure of radioactive iodine therapy (RIT) in GD patients (19).Besides, emerging evidence from other autoimmune diseases has revealed a close link between VitD dynamics and glucocorticoid therapeutic efficacy. In patients with severe systemic lupus erythematosus receiving IVGC therapy and VitD supplementation, the increase in serum VitD concentrations was predictive of complement levels normalization (20). However, the relationship between VitD and both the onset and treatment response of TAO remains incompletely elucidated. Crucially, due to the counteraction of osteoporosis risk induced by IVGC, the routine supplementation of VitD and calcium was given during the therapy, which makes the relationship more complex and interesting.

To better explore the clinical association between VitD and TAO, patients who completed the IVGC therapy course were enrolled in the study and grouped based on their response to the treatment. Baseline, post-treatment and pre-post treatment changes were all analyzed to fully demonstrate the role of VitD and its changes on the treatment response of TAO. This research aims to provide a new insight for further studies on pathogenesis and clinical management for TAO.

## 2 Materials and methods

### 2.1 Study design and participants

**Figure 1:**
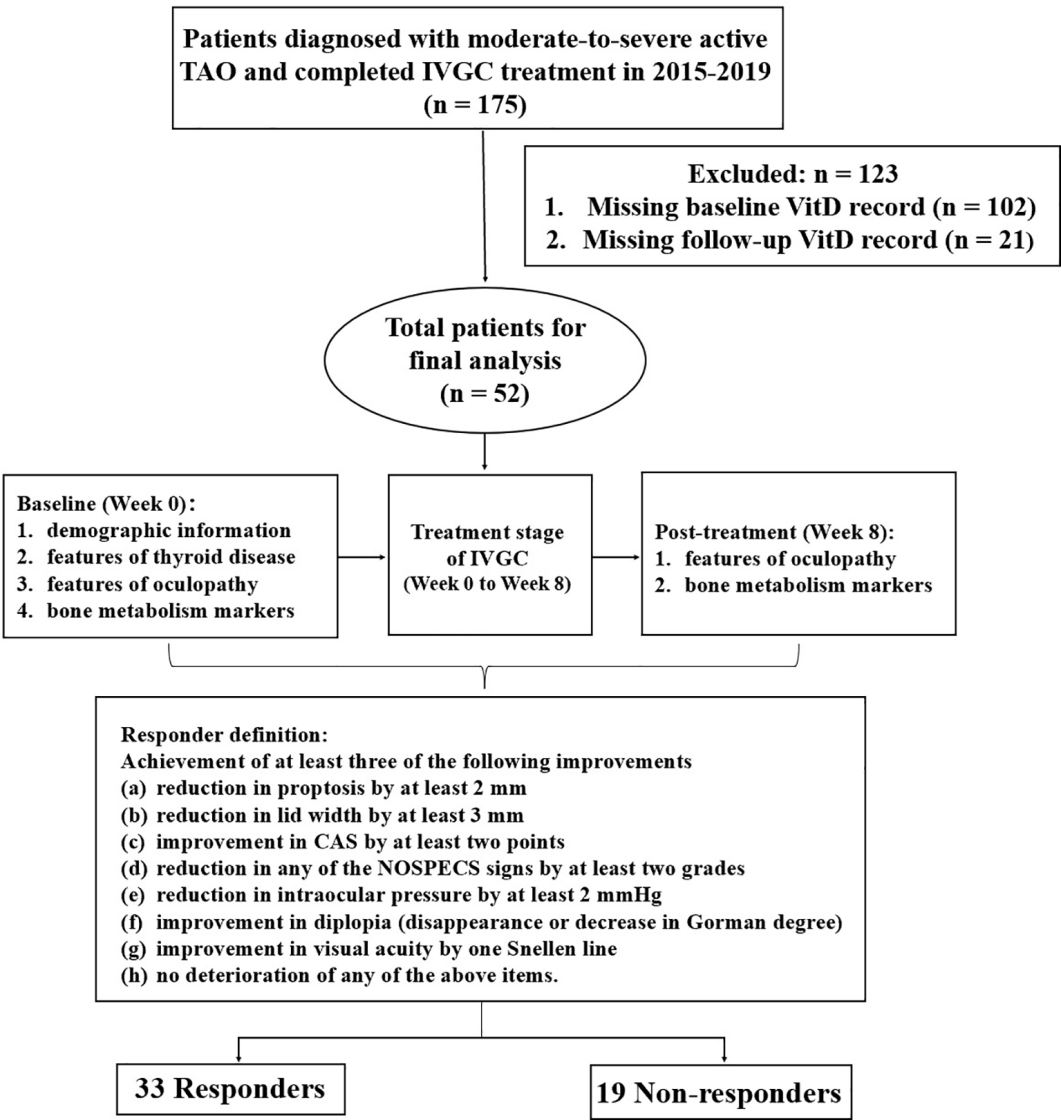
Study flow chart. TAO, thyroid-associated ophthalmopathy; IVGC, intravenous glucocorticoids; VitD, vitamin D; CAS, clinical activity score.

We conducted a retrospective study of consecutive patients who were diagnosed with moderate-to-severe, active TAO and completed the full course of IVGC at the Department of Endocrinology, First Affiliated Hospital of Xi’an Jiaotong University, from January 2015 to December 2019. All patients were over 18 years old and were diagnosed with TAO by a professional clinician based on clinical manifestations and the orbital CT results. Patients without baseline VitD or follow-up VitD were excluded from this study (**Figure 1**). This study was approved by the Institutional Review Board of the First Affiliated Hospital of Xi’an Jiaotong University (XJTU1AF2024LSYY-096) and was in line with the principles of the Helsinki Declaration on Clinical Research.

The treatment protocol of IVGC was as follows: 0.5–1.0 g of intravenous methylprednisolone was given every other day three times and was repeated at 3-week intervals for a total of 3 cycles. The choice of 500 mg or 1.0 g IVGC was determined by disease activity, severity, and the presence of any relative contraindications. The cumulative dose after the treatment ranged from 4.5 g to 9.0 g. During the course of treatment, all patients were prescribed calcium and VitD supplements. Proton pump inhibitors or H2 receptor antagonists were also prescribed. Antithyroid agents or thyroxine were used to restore and maintain euthyroidism.

### 2.2 Ophthalmic assessments and outcome evaluation

Ophthalmic assessments included the presence of diplopia, the clinical activity score (CAS) for disease activity, the NOSPECS score (no TAO sign, only eyelid sign, soft tissue involvement, proptosis, extraocular motility restriction, corneal involvement, sight loss) for disease severity, visual acuity (VA), intraocular pressure (IOP), and exophthalmometry (21). In particular, the E Standard Logarithmic Visual Acuity Chart (GB 11533-1989) was used to measure VA at a distance of 5 m (22). The corresponding relationship between the E chart and the Snellen chart is as follows: 0.1, 0.2, 0.4, 0.8, and 1 in the E chart are equivalent to 20/200, 20/100, 20/50, 20/25, and 20/20 in the Snellen chart, respectively. Considering the possibility of a unilateral onset of TAO, for patients with bilateral data for vision, IOP, and exophthalmometry, the more severe values were used.

Results of ophthalmic assessments before and after IVGC therapy were compared to evaluate therapeutic outcomes, patients were divided into “responders” and “non-responders”. “Responsive” was defined as the achievement of at least three of the following improvements (23): (a) reduction in proptosis by at least 2 mm, (b) reduction in lid width by at least 3 mm, (c) improvement in CAS by at least two points, (d) reduction in any of the NOSPECS signs by at least two grades, (e) reduction in intraocular pressure by at least 2 mmHg, (f) improvement in diplopia (disappearance or decrease in Gorman degree), (g) improvement in visual acuity by one Snellen line and (h) no deterioration of any of the above items.

### 2.3 The collection of variates

The list of TAO patients receiving IVGC therapy was retrieved from the medical records system of the First Affiliated Hospital of Xi’an Jiaotong University. Basic information, anthropometric measurements, medication history, and laboratory data were collected.

Basic information included age, sex, and smoking status. The medication history included the duration of thyroid disease, the usage of anti-thyroid drugs and radioactive iodine therapy, the duration of ophthalmopathy. The anthropometric indicators included systolic blood pressure (SBP), diastolic blood pressure (DBP), body mass index (BMI), and ophthalmic assessments described above. The laboratory examination results included metabolism indicators, thyroid functions, and bone metabolism markers. Venous blood samples were collected in the morning after an 8-hour fast. Biochemical analyses, including fasting blood glucose (FBG), total cholesterol (TC), triglycerides (TG), high-density lipoprotein cholesterol (HDL-c), low-density lipoprotein cholesterol (LDL-c), calcium (Ca), phosphorus (P), and alkaline phosphatase (ALP), were performed using a LABOSPECT 008AS automatic biochemical analyzer (HITACHI, Japan). 25-hydroxyvitamin D (25(OH)D), parathyroid hormone (PTH), N-mid osteocalcin (N-MID OC), beta-cross-linked C-telopeptide of type I collagen (β-CTX), and total procollagen type I N-terminal propeptide (tPINP) were measured using a Cobas 8000 E602 automatic analyzer (Roche, Germany). Free triiodothyronine (FT3), free thyroxine (FT4), thyrotropin (TSH), triiodothyronine (T3), thyroxine (T4), thyroglobulin antibodies (TGAb), thyroid microsomal antibodies (TMAb), and thyroid peroxidase antibody (TPOAB) were measured by a GC-1200 gamma radioimmunoassay counter (CNNC, Anhui, China). Glycated hemoglobin (HbA1c) was measured via an HLC-723G8 automatic analyzer (TOSOH BIOSCIENCE, Japan).

### 2.4 Statistical analysis

Statistical analysis was performed using SPSS 25.0 software. A two-tailed *P* value < 0.05 was considered statistically significant.

Continuous variables were tested for normality using the Shapiro-Wilk test. Normally distributed variables were presented as mean ± standard deviation (SD), non-normally distributed continuous variables were presented as median (interquartile range, IQR), and categorical variables were presented as number (percentage, %). For inter-group comparisons between IVGC responders and non-responders, independent samples Student’s t-test was used for normally distributed data, Mann–Whitney U test for non-normally distributed data, and Pearson’s chi-square test or Fisher’s exact test for categorical data. Pre-post treatment changes were calculated as (post-treatment value minus baseline value) and expressed as delta (Δ), and inter-group differences in Δ values were compared using the same inter-group test rules above. For within-group paired comparisons (baseline vs. post-treatment), paired samples t-test and Wilcoxon signed-rank test were applied for normally and non-normally distributed data, respectively.

Univariate binary logistic regression was performed to assess the association between each candidate variable and IVGC treatment response, with odds ratios (ORs) and 95% confidence intervals (CIs) calculated. Multivariate binary logistic regression was further conducted to identify independent predictors of IVGC response, with variables showing a *P* value < 0.05 in the baseline inter-group comparison, as well as clinically related indicators included as confounding factors for adjustment.

The receiver operating characteristic curve (ROC) analysis, with the area under the curve (AUC) calculated was used to calculate the discriminatory performance of Δ25(OH)D for IVGC efficacy. *Post-hoc* power analysis was also conducted to assess the actual statistical power of this study.

## 3 Results

### 3.1 Baseline characteristics of the study participants

A total of 52 TAO patients who completed the IVGC therapy course and had both pre- and post-treatment VitD measurements were included (**Figure 1**). Of these, 33 patients responded to treatment (responders) and 19 did not (non-responders), yielding an IVGC treatment response rate of 63.46%. This response rate aligns with previous studies (9).

Baseline clinical characteristics of the two groups are summarized in **Table 1**. There were no significant differences between the two groups in terms of gender, age, smoking status, most metabolic parameters and characteristics of thyroid diseases. Among metabolic parameters, TG levels were significantly higher in non-responders compared to responders (*P* = 0.019). For clinical features of thyroid disease, non-responders had significantly higher T4 levels (*P* = 0.010).

**Table 1:** Baseline characteristics of the study participants.

Variables	Total	Responders	Non-responders	*P* value
	(N = 52)	(N = 33)	(N = 19)	
Male (%)	24 (46.15)	13 (39.39)	11 (57.89)	0.198
Age (years)	48.60 ± 12.28	48.82 ± 13.49	48.21 ± 10.16	0.865
Smoking status (%)				0.325
Non-smokers	36 (69.23)	25 (75.76)	11 (57.89)	
Ex-smokers	3 (5.77)	1 (3.03)	2 (10.53)	
Current smokers	13 (25.00)	7 (21.21)	6 (31.58)	
BMI (kg/m^2^)	23.14 ± 3.36	22.38 ± 3.07	24.31 ± 3.54	0.072
SBP(mmHg)	120.37 ± 9.59	119.33 ± 9.72	122.12 ± 9.35	0.321
DBP(mmHg)	70.84 ± 7.54	69.54 ± 7.95	73.04 ± 6.40	0.110
FBS (mmol/L)	4.56 (4.26-5.19)	4.52 (4.29-5.46)	4.59 (4.24-4.73)	0.279
HbA1c (%)	5.34 ± 0.45	5.32 ± 0.50	5.39 ± 0.34	0.594
TG (mmol/L)	1.16 (0.86-1.72)	1.04 (0.80-1.42)	1.34 (1.11-2.34)	**0.019**
TC (mmol/L)	3.99 (3.41-4.63)	3.86 (3.29-4.73)	4.45 (3.54-4.63)	0.203
LDL-c (mmol/L)	2.34 (1.88-2.99)	2.15 (1.76-2.81)	2.62 (2.05-3.10)	0.123
HDL-c(mmol/L)	1.14 (0.93-1.42)	1.18 (0.99-1.42)	1.03 (0.82-1.43)	0.366
Thyroid Dysfunction (%)				0.400
Euthyroid	1 (1.92)	1 (3.03)	0 (0)	
Hyperthyroidism	49 (94.23)	30 (90.91)	19 (100)	
Hypothyroidism	2 (3.85)	2 (6.06)	0 (0)	
Duration of Thyroid disease (months)	10.00 (5.00-12.00)	9.00 (5.00-12.00)	10.00 (6.00-20.00)	0.445
ATD (%)				0.331
Imidazoles (%)	38 (73.08)	24 (72.73)	14 (73.68)	
Thioureas (%)	1 (1.92)	1 (3.03)	0 (0)	
Others (%)	3 (5.77)	3 (9.09)	0 (0)	
RAI (%)	4 (7.69)	2 (6.06)	2 (10.50)	0.561
Positive TPOAb (%)	31 (59.62)	22 (66.67)	9 (47.37)	0.274
Positive TGAb (%)	3 (5.77)	3 (9.09)	0 (0)	0.205
Positive TMAb (%)	12 (23.08)	9 (27.27)	3 (15.79)	0.322
T4 (ug/dL)	8.12 (6.44-13.20)	8.97 (7.13-18.50)	7.06 (5.71-8.32)	**0.010**
T3 (ng/mL)	1.46 (1.21-2.07)	1.50 (1.27-2.20)	1.35 (1.13-1.81)	0.470
FT4 (pmol/L)	16.00 (13.59-17.85)	15.90(13.70-20.00)	16.15(12.58-17.45)	0.507
FT3 (pmol/L)	5.47 (4.75-6.08)	5.40 (4.70-6.07)	5.70 (4.79-6.12)	0.619
TSH (uIU/mL)	0.39 (0.07-3.09)	0.32 (0.07-3.94)	0.98 (0.07-3.65)	0.562

BMI, body mass index; SBP, systolic blood pressure; DBP, diastolic blood pressure; FBS, fasting blood sugar; HbA1c, glycated hemoglobin; TG, triglyceride; TC, total cholesterol; LDL-c, low-density lipoprotein cholesterol; HDL-c, high-density lipoprotein cholesterol; ATD, anti-thyroid drugs; RAI, radioactive iodine therapy; TPOAb, thyroid peroxidase antibodies; TGAb, thyroglobulin antibodies; TMAb, thyroid microsomal antibodies; T4, thyroxine; T3, triiodothyronine; FT4, free thyroxine; FT3, free triiodothyronine; TSH, thyrotropin. Continuous variables are presented as mean ± standard deviation (SD) for normally distributed data, or median (interquartile range, IQR) for non-normally distributed data. Categorical variables are presented as number (percentage, %). Independent samples t-test was used for inter-group comparison of normally distributed continuous variables, Mann–Whitney U test for non-normally distributed continuous variables, and Pearson’s chi-square test (or Fisher’s exact test where appropriate) for categorical variables. The bold *P* value indicated statistical significance (*P* < 0.05).

### 3.2 Ophthalmic outcome evaluation of IVGC therapy

To demonstrate the therapeutic outcomes of IVGC, baseline and pre-post treatment changes (Δ) of the ophthalmic features are presented in **Table 2**. Non-responders had a significantly longer duration of ophthalmopathy than responders at baseline (*P* = 0.003). No significant differences were observed between the two groups in baseline CAS, NOSPECS score, diplopia prevalence, visual acuity, exophthalmometry, or IOP (*P*>0.05). After the IVGC therapy, responders demonstrated significantly greater improvement compared to non-responders. Specifically, responders showed a significantly greater reduction in CAS (*P* = 0.002) and a significantly greater improvement in NOSPECS score (*P* = 0.047). In addition, the diplopia relief rate was significantly higher in responders than in non-responders (*P* = 0.025).

**Table 2:** Ophthalmic outcome evaluation of IVGC therapy.

Variables	Total	Responders	Non-responders	*P* value
	(N = 52)	(N = 33)	(N = 19)	
Baseline				
Duration of oculopathy (months)	4.50 (3.00-8.75)	3.00 (2.50-6.00)	8.00 (4.00-12.00)	**0.003**
CAS	4.00 (3.00-5.00)	4.00 (3.00-5.00)	3.00 (2.00-5.00)	0.106
NOSPECS score	4.00 (4.00-4.00)	4.00 (4.00-4.50)	4.00 (4.00-4.00)	0.327
Diplopia (%)	35 (67.31)	23 (69.70)	12 (63.16)	0.824
VA	0.60 (0.30-0.90)	0.60 (0.30-0.80)	0.50 (0.30-1.00)	0.597
Exophthalmometry (mm)	18.00 (16.00-19.00)	17.00 (15.00-19.00)	18.00 (17.00-20.00)	0.163
IOP (mmHg)	17.92 ± 3.96	17.96 ± 4.23	17.86 ± 3.64	0.935
Pre-post treatment Changes (Δ)				
Δ CAS	-2.00 (-3.00 to -1.00)	-2.00 (-3.00 to -1.00)	-1.00 (-1.00 to 0.00)	**0.002**
Δ NOSPECS score	0.00 (0.00 to 0.00)	0.00 (0.00 to 0.00)	0.00 (0.00 to 0.00)	**0.047**
Diplopia relief, n (%) ^a^	21 (40.38)	17 (51.52)	4 (21.05)	**0.025**
Δ VA	0.00 (-0.08 to 0.20)	0.10 (0.00 to 0.30)	0.00 (-0.10 to -0.15)	0.208
Δ Exophthalmometry (mm)	-0.16 ± 1.91	0.19 ± 1.89	-0.59 ± 1.91	0.216
Δ IOP (mmHg)	0.00 (-1.00 to 2.00)	0.00 (-1.63 to 1.13)	1.00 (-0.25 to 2.00)	0.108

CAS, clinical activity score; VA, visual acuity; IOP, intraocular pressure. Continuous variables are presented as mean ± standard deviation (SD) for normally distributed data, or median (interquartile range, IQR) for non-normally distributed data. Categorical variables are presented as number (percentage, %). Independent samples t-test was used for inter-group comparison of normally distributed continuous variables, Mann–Whitney U test for non-normally distributed continuous variables, and Pearson’s chi-square test (or Fisher’s exact test where appropriate) for categorical variables. The bold *P* value indicated statistical significance (*P* < 0.05). Δ: change calculated as post-treatment value minus baseline value. ^a^Relief rate of diplopia after treatment in patients with baseline diplopia.

### 3.3 Dynamic changes in 25(OH)D levels and bone metabolism markers during the IVGC therapy

Subsequently, we investigated the changes in 25(OH)D and bone metabolism markers during treatment (**Table 3**). For baseline parameters, β-CTX and N-MID OC levels were significantly higher in responders compared to non-responders (*P* = 0.006 and *P* = 0.015, respectively), but there was no difference in 25(OH)D between the two groups (*P*>0.05).

**Table 3:** Dynamic changes in 25(OH)D levels and bone metabolism markers during the IVGC therapy.

Variables	Total	Responders	Non-responders	*P* value
	(N = 52)	(N = 33)	(N = 19)	
Baseline				
25(OH)D (ng/mL)	14.95 (10.10-21.48)	13.60 (8.65-19.10)	17.50 (14.10-25.70)	0.055
25(OH)D deficiency (≤20ng/mL, %)	38 (73.08)	27 (81.82)	11 (57.89)	0.061
Ca (mmol/L)	2.19 (2.12-2.27)	2.19 (2.10-2.28)	2.18 (2.14-2.27)	0.842
P (mmol/L)	1.16 ± 0.21	1.10 ± 0.19	1.20 ± 0.22	0.110
ALP (U/L)	99.00 (76.00-136.75)	109.00 (74.00-152.50)	97.00 (79.00-114.00)	0.328
PTH (pg/mL)	42.15 (31.60-60.43)	41.30 (31.55-56.85)	42.20 (31.50-68.20)	0.642
N-MID OC (ng/mL)	35.60 (25.53-47.50)	40.60 (30.55-50.85)	29.30 (20.70-39.60)	**0.015**
β-CTX (pg/mL)	958.85 (716.58-1350.75)	1049.00 (864.40-1540.50)	832.60 (602.20-1043.00)	**0.006**
tPINP (ng/mL)	118.90 (80.84-205.70)	142.40 (95.94-239.45)	89.77 (46.18-141.50)	0.050
Pre-post Treatment Changes (Δ)				
Δ 25(OH)D (ng/mL)	1.08 ± 4.83	2.41 ± 4.99	-1.22 ± 3.60	**0.008**
Δ Ca(mmol/L)	-0.04 (-0.12 to 0.08)	-0.06 (-0.14 to 0.07)	-0.01 (-0.10 to 0.20)	0.259
Δ P(mmol/L)	-0.07 ± 0.21	-0.09 ± 0.19	-0.02 ± 0.23	0.252
Δ ALP (U/L)	-10.00 (-24.00 to 0.00)	-9.50 (-26.00 to 5.25)	-11.00 (-24.00 to 3.00)	0.360
Δ PTH (pg/mL)	6.59 ± 20.92	6.10 ± 22.42	7.52 ± 18.50	0.834
Δ N-MID OC (ng/mL)	-3.00 (-9.73 to 2.98)	-3.10 (-10.10 to 4.70)	-2.40 (-6.30 to -0.40)	0.916
Δ β-CTX (pg/mL)	-201.65 (-475.00 to -44.83)	-256.90 (-514.00 to -71.50)	-94.00 (-404.50 to -35.00)	0.254
Δ tPINP (ng/mL)	-10.16 (-37.14 to 8.88)	-8.60 (-25.30 to 10.82)	-31.25 (-62.03 to 3.60)	0.103

25(OH)D, 25-hydroxyvitamin D; Ca, calcium; P, phosphorus; ALP, alkaline phosphatase; PTH, parathyroid hormone; N-MID OC, N-terminal mid-fragment osteocalcin; β-CTX, β-CrossLaps; tPINP, total procollagen type I N-terminal propeptide. Continuous variables are presented as mean ± standard deviation (SD) for normally distributed data, or median (interquartile range, IQR) for non-normally distributed data. Categorical variables are presented as number (percentage, %). Independent samples t-test was used for inter-group comparison of normally distributed continuous variables, Mann–Whitney U test for non-normally distributed continuous variables, and Pearson’s chi-square test (or Fisher’s exact test where appropriate) for categorical variables. The bold P value indicated statistical significance (*P* < 0.05). Δ: change calculated as post-treatment value minus baseline value.

**Figure 2:**
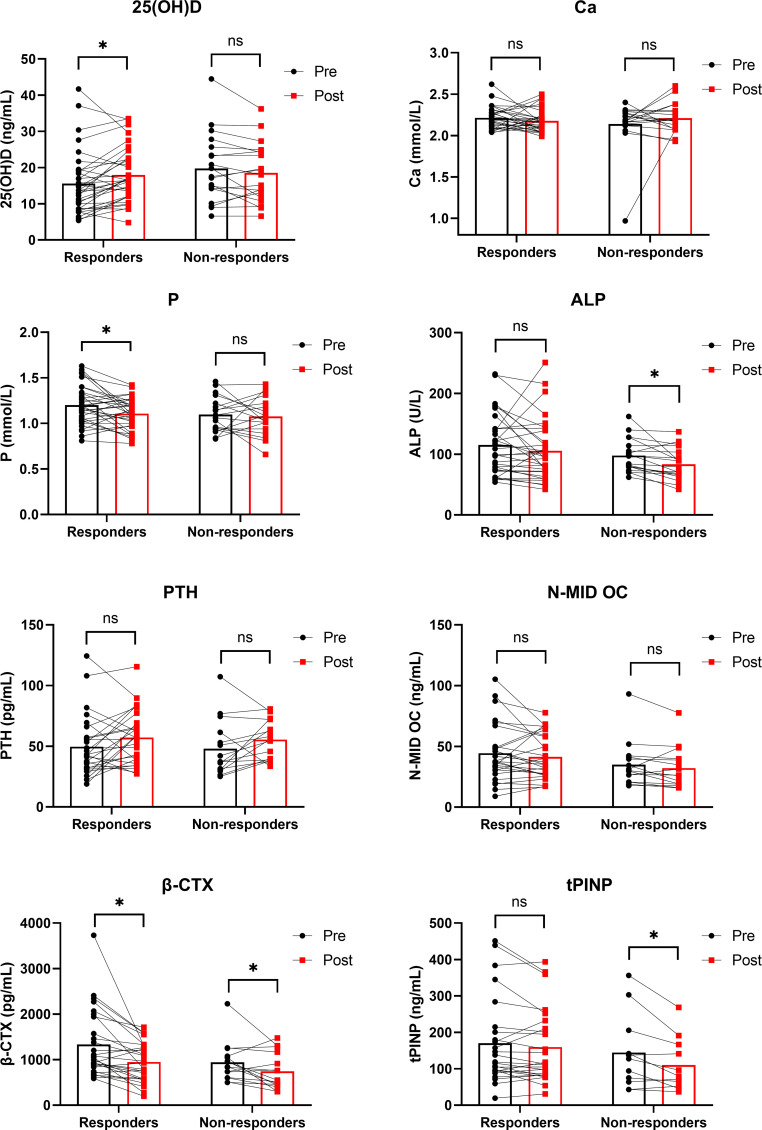
Within-group changes in 25(OH)D levels and bone metabolism markers from pre-treatment to post-treatment. 25(OH)D, 25-hydroxyvitamin D; Ca, calcium; P, phosphorus; ALP, alkaline phosphatase; PTH, parathyroid hormone; N-MID OC, N-terminal mid-fragment osteocalcin; β-CTX, β-CrossLaps; tPINP, total procollagen type I N-terminal propeptide. Within-group comparisons (pre vs. post) were performed using paired samples t-test or Wilcoxon signed-rank test. *P < 0.05.

**Figure 3:**
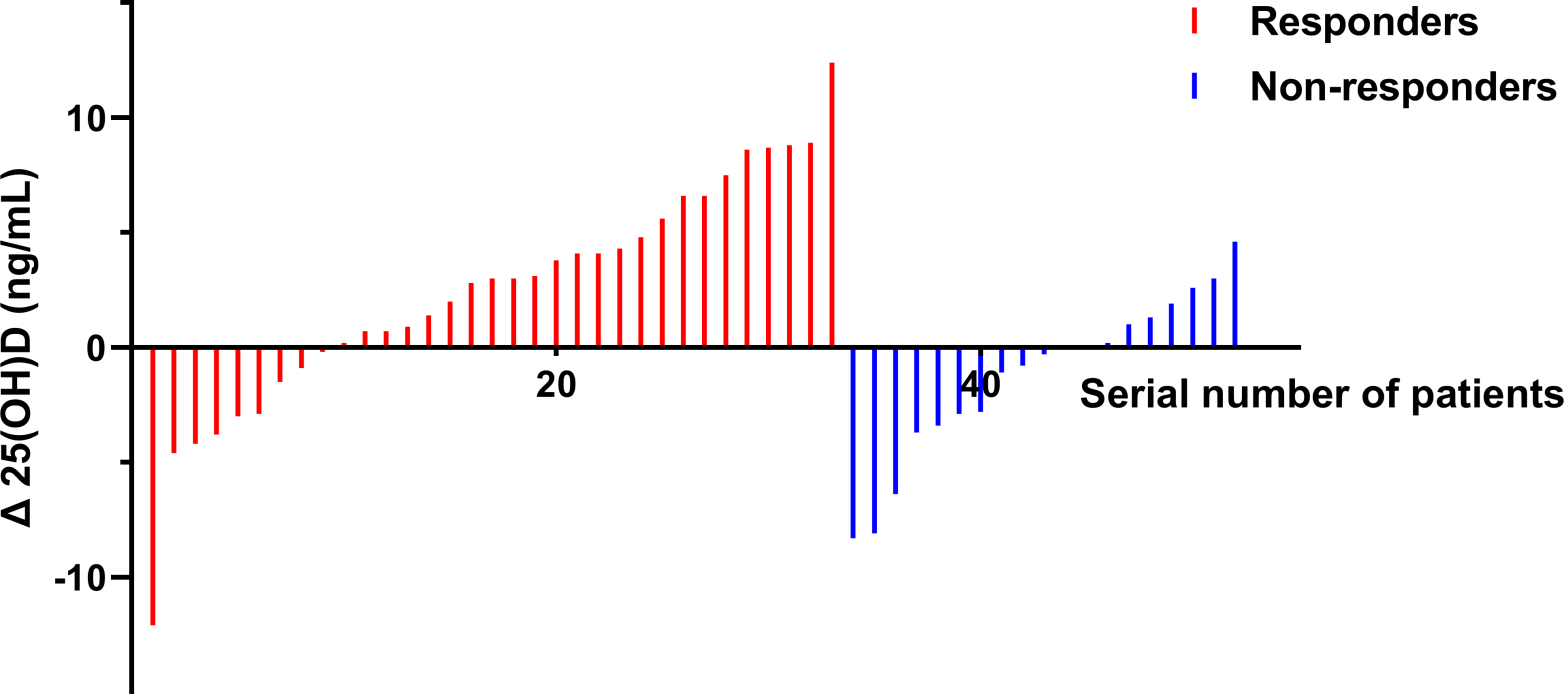
Changes in 25(OH)D Levels from Baseline to Post-treatment in Responders and Non-responders. Δ25(OH)D, change in serum 25-hydroxyvitamin D level. Red bars represent responders (n=33), blue bars represent non-responders (n=19).

To explore within-group changes in 25(OH)D levels and bone metabolism markers before and after treatment, we plotted **Figure 2**. After treatment, the responders exhibited a significant increase in 25(OH)D (*P* = 0.02), but there was no change in the non-responders (*P*>0.05). Moreover, significant decreases in β-CTX levels were observed in both responders (*P* = 0.001) and non-responders (*P* = 0.031), significant decreases in P levels were observed in responders (*P* = 0.01), and significant decreases in ALP and tPINP levels were observed in non-responders (*P* = 0.005 and *P* = 0.019, respectively). We further analyzed pre-post treatment changes (Δ) in 25(OH)D levels and bone metabolism markers (**Table 3**). Of note, the increase in 25(OH)D was significantly greater in responders than in non-responders (*P* = 0.008). We further visualized the individual-level Δ25(OH)D in responders and non-responders (**Figure 3**).

### 3.4 Univariate logistic regression analysis of bone metabolism markers on the efficacy of IVGC

**Figure 4:**
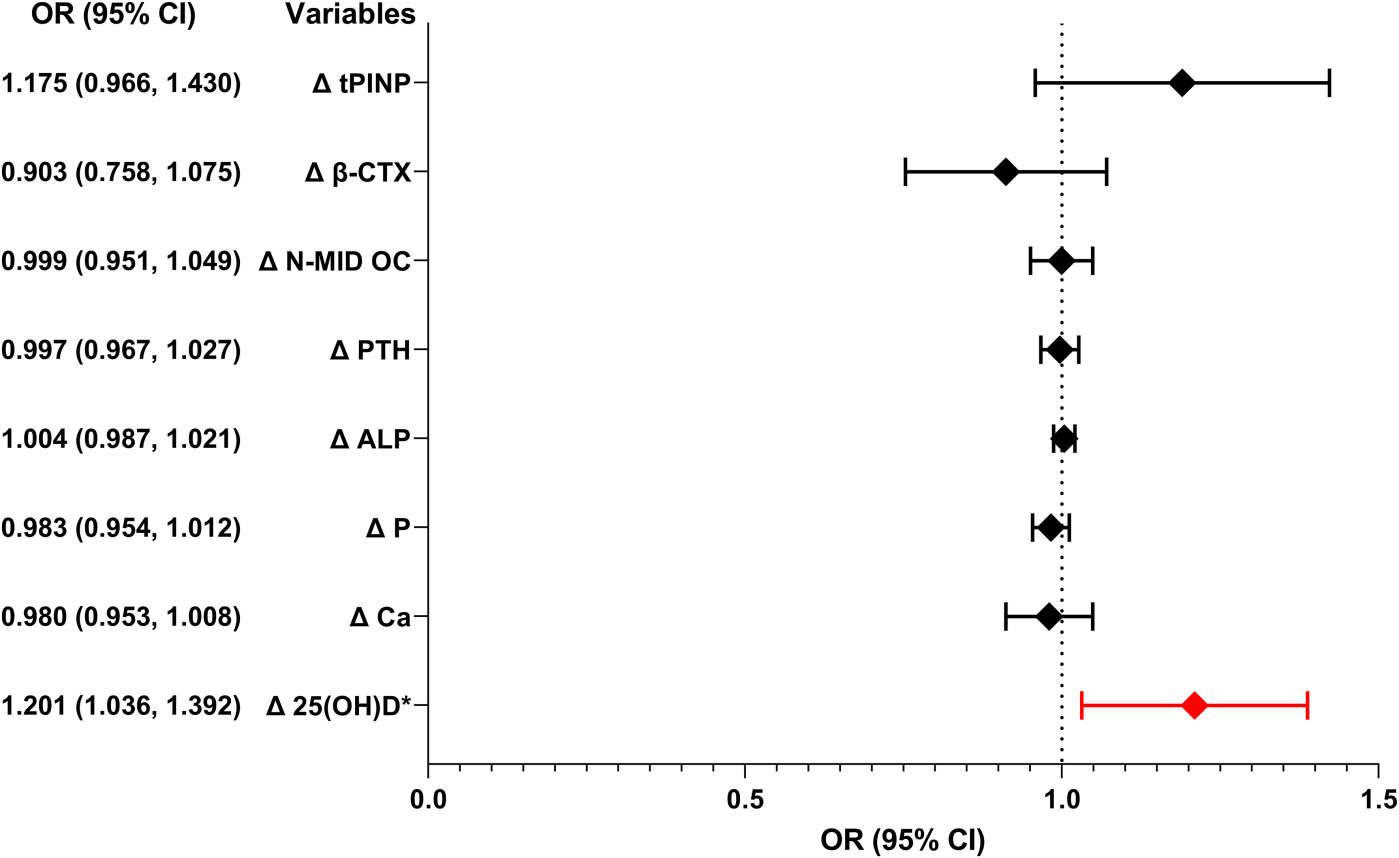
Forest graph showing the results of univariate logistic regression analyses of the association between the changes of bone metabolism markers and the efficacy of IVGC. OR, odds ratio; CI, confidence interval; tPINP, total procollagen type I N-terminal propeptide; β-CTX, β-CrossLaps; N-MID OC, N-terminal mid-fragment osteocalcin; PTH, parathyroid hormone; ALP, alkaline phosphatase; P, phosphorus; Ca, calcium; 25(OH)D, 25-hydroxyvitamin D. Δ: change calculated as post-treatment value minus baseline value. *P < 0.05.

To further investigate the relationship between bone metabolism markers and the efficacy of IVGC in TAO, univariate logistic regression analyses were conducted. As shown in **Figure 4**, Δ25(OH)D emerged as the most significant positive predictor of the efficacy of IVGC (odds ratio (OR): 1.201, 95% confidence interval (CI): 1.036-1.392, *P* = 0.015). The baseline β-CTX levels also showed a significant positive predictor of the efficacy of IVGC (OR: 1.197, 95% CI: 1.020-1.406, *P* = 0.028) (**Supplementary Figure 1**).

### 3.5 Multivariate logistic regression analysis of the independent effect of Δ25(OH)D on the efficacy of IVGC

To account for confounding factors, variables with a *P* value < 0.05 in the baseline inter-group comparison, as well as clinically related indicators, were included in the multivariate binary logistic regression models to verify the independent effects of Δ25(OH)D on the efficacy of IVGC. After adjusting for age and gender, a positive correlation was found between them in the basic model (OR: 1.205, 95% CI: 1.033-1.405, *P* = 0.017). *P* values and ORs were not strongly affected when other possible confounders, such as the TG, duration of ophthalmopathy, baseline 25(OH)D and baseline β-CTX, were accounted for independently on the basis of the basic model (**Table 4**).

**Table 4:** Multivariate logistic regression analysis of the independent effect of Δ25(OH)D on the efficacy of IVGC.

Models	Adjusted for	OR (95% CI)	*P* value
Model 1	age, Gender	1.205 (1.033, 1.405)	**0.017**
Model 2	age, Gender, TG	1.219 (1.029, 1.444)	**0.022**
Model 3	age, Gender, TC	1.221 (1.040, 1.432)	**0.015**
Model 4	age, Gender, T4	1.432 (1.118, 1.833)	**0.004**
Model 5	age, Gender, T3	1.310 (1.088, 1.578)	**0.004**
Model 6	age, Gender, FT3	1.267 (1.067, 1.503)	**0.007**
Model 7	age, Gender, FT4	1.326 (1.087, 1.617)	**0.006**
Model 8	age, Gender, TSH	1.206 (1.032, 1.408)	**0.018**
Model 9	age, Gender, Duration of oculopathy	1.298 (1.061, 1.588)	**0.011**
Model 10	age, Gender, 25(OH)D	1.195 (1.002, 1.424)	**0.047**
Model 11	age, Gender, N-MID OC	1.242 (1.043, 1.478)	**0.015**
Model 12	age, Gender, β-CTX	1.208 (1.018, 1.433)	**0.030**

Δ25(OH)D, change in serum 25-hydroxyvitamin D level; IVGC, intravenous glucocorticoid; OR, odds ratio; CI, confidence interval; TG, triglyceride; TC, total cholesterol; T4, thyroxine; T3, triiodothyronine; FT4, free thyroxine; FT3, free triiodothyronine; TSH, thyrotropin; N-MID OC, N-terminal mid-fragment osteocalcin; β-CTX, β-CrossLaps. Multivariate logistic regression analysis was performed, with Δ25(OH)D as the independent variable and IVGC treatment response as the dependent variable. Models were adjusted for age, gender, and other potential confounding factors. The bold P value indicated statistical significance (*P* < 0.05).

### 3.6 Receiver operating characteristic analysis to determine the power of Δ25(OH)D on the efficacy of IVGC

**Figure 5:**
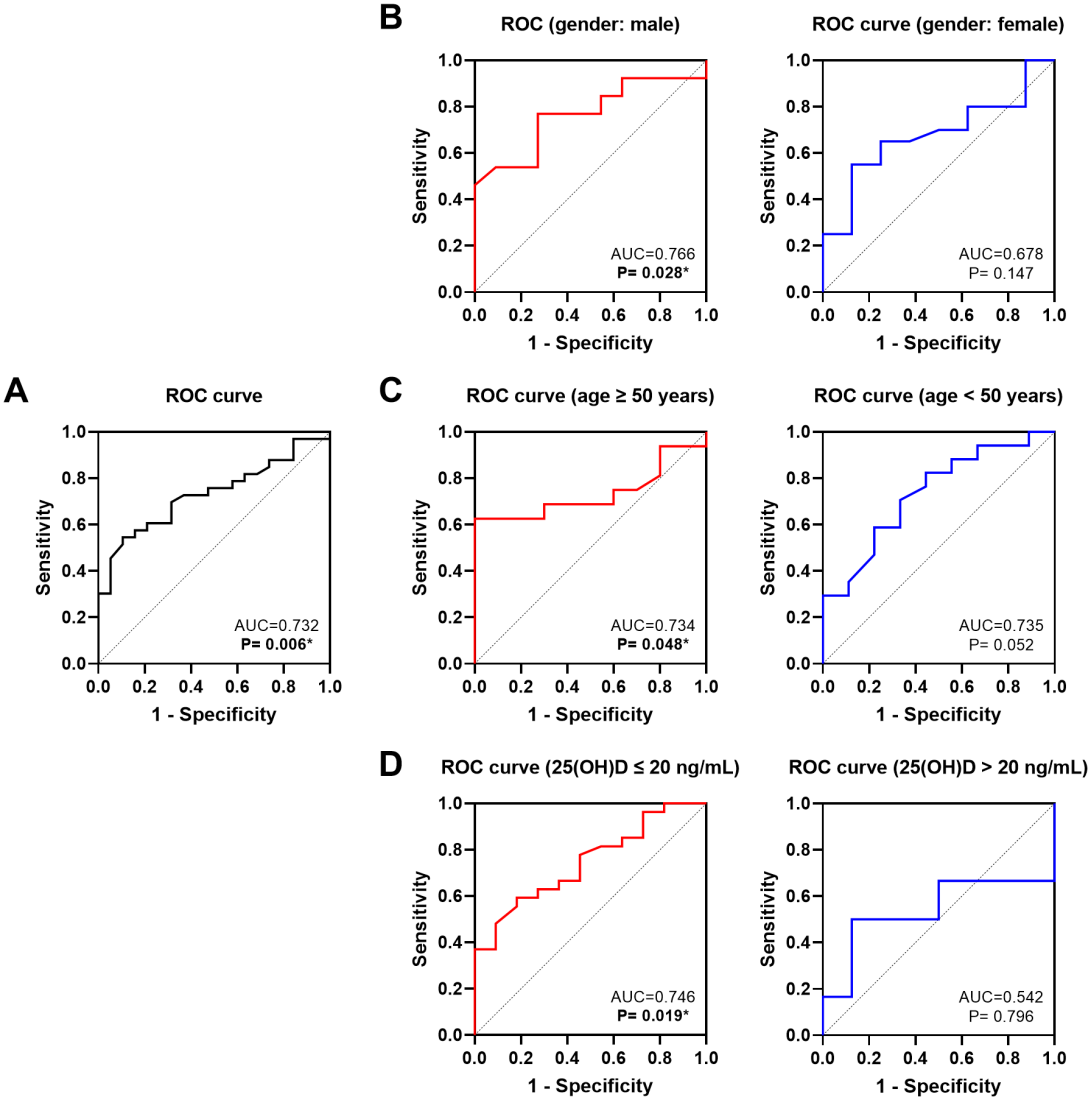
ROC analysis of the effect of the Δ25(OH)D on the efficacy of IVGC in. **(A)** all patients; **(B)** patients of different genders; **(C)** patients aged ≥50 years or <50 years; **(D)** patients with baseline vitamin D levels ≤20 ng/mL or >20 ng/mL. ROC, receiver operating characteristic; AUC, area under the curve. 25(OH)D, 25-hydroxyvitamin **(D)** Δ: change calculated as post-treatment value minus baseline value. *P < 0.05.

Considering that a higher Δ25(OH)D is an independent positive factor for the efficacy of IVGC, we performed an ROC analysis (**Figure 5A**). The analysis yielded an AUC of 0.732, with a Youden index of 0.440(*P* = 0.006, optimal cut-off value=2.7).

We further performed subgroup ROC analyses stratified by different gender, age and baseline 25(OH)D statuses (**Figures 5B–D**). The results demonstrated that Δ25(OH)D had a stronger predictive value for the efficacy of IVGC on TAO in male patients (AUC = 0.766, Youden index=0.497, *P* = 0.028, optimal cut-off value=0.35), older patients (≥50 years, AUC = 0.766, Youden index=0.625, *P* = 0.025, optimal cut-off value=2.7) and patients with 25(OH)D deficiency (AUC = 0.746, Youden index=0.411, *P* = 0.019, optimal cut-off value=2.7).

### 3.7 *Post-hoc* power analysis

To assess the reliability of the observed intergroup difference in Δ25(OH)D, a *post-hoc* power analysis was conducted using SPSS 25.0 software. The sample sizes of the responders and non-responders were 33 and 19, respectively. The mean ± SD of the two groups were 2.41 ± 4.99 and -1.22 ± 3.60, respectively. After setting the inspection direction to two-tailed test and α to 0.05, the statistical power was 84.3%. This high power (>80%) indicates that the study had sufficient sensitivity to detect the significant difference in Δ25(OH)D between responders and non-responders.

## 4 Discussion

This retrospective study showed a 73.08% prevalence of 25(OH)D deficiency in TAO patients, and only responders had a significant 25(OH)D increase. Changes in 25(OH)D levels during therapy (Δ25(OH)D), not baseline levels, independently predicted IVGC response in TAO patients, with stronger effects in males, the elderly, and VitD -deficient individuals. These findings emphasize the need to monitor and manage VitD supplementation during IVGC therapy to early identify non-responders and improve efficacy.

Our study identified ΔVitD, rather than baseline VitD levels, as the key factor influencing the efficacy of IVGC therapy in TAO patients. While numerous studies implicate VitD in GD pathogenesis, evidenced by a higher prevalence of VitD deficiency in GD patients compared to healthy individuals (17, 24) and its association with suboptimal responses to GD treatments like ATD or RIT (18, 19), research specifically on TAO suggests baseline VitD may not play a significant role. Specifically, baseline VitD levels in TAO patients show no significant difference from GD patients without ocular involvement (25), and VitD deficiency in TAO is not associated with disease severity or activity (26). Consistent with this, our study found no association between baseline VitD levels and IVGC therapeutic outcome in TAO. These findings collectively indicate that baseline VitD levels lack a significant correlation with TAO onset or progression, which may be attributed to multiple confounding factors, including seasonal variation, sunlight exposure, dietary intake, and underlying disease status (27).

It is well-established that glucocorticoids reduce VitD levels (28, 29). Consequently, VitD levels following supplementation exhibit complex dynamics during IVGC therapy, with not all patients showing an increase (30). In this context, ΔVitD may more accurately reflect an individual’s response to supplementation and the dynamic interaction between VitD and glucocorticoid therapy, offering advantages over a single baseline measurement. We identified Δ25(OH)D as an independent predictor of IVGC response in TAO patients, with a cut-off value of +2.7 ng/mL. This finding aligns with evidence from other diseases, where dynamic changes in VitD levels have proven superior to static measurements in predicting prognosis. A prospective study on polymyalgia rheumatica found that although baseline VitD levels were not associated with disease activity, ΔVitD was the strongest independent factor predicting patient remission after 3-month VitD supplementation therapy (OR = 2.89; 95% CI 1.60-4.11), with significantly higher increases in VitD levels in responders compared to non-responders (+22.02 vs.+1.33 ng/mL, *P* = 0.044) *(*31). Mechanistically, ΔVitD may more accurately reflect the functional capacity of the VitD axis during GC therapy than static baseline levels, as it represents the dynamic response to VitD repletion.

The potential mechanisms underlying the predictive value of ΔVitD for IVGC efficacy mainly involve the synergistic anti-inflammatory and immunomodulatory effects of VitD and glucocorticoids. VitD primarily exerts its immunoregulatory function, particularly on T cell immunity, through the VitD receptor (VDR) on cell membranes (32). Naïve T cells express relatively low levels of VDR on their surface, but receptor expression gradually increases upon activation (33). Studies demonstrate that VitD can suppress the activation, proliferation, and differentiation of T cells (33). VitD triggered the contraction of T-helper (Th)1 by recruiting several transcription factors, notably c-JUN, STAT3 and BACH2, and then initiated the transition from pro-inflammatory interferon-γ+ Th1 cells to suppressive interleukin-10+ regulatory T cells (Tregs) (34). VitD could also promote the Th2 polarization through a STAT6/GATA3 way (35). Moreover, VitD inhibited the differentiation and expansion of Th17 cells from both naïve and memory T cells by suppressing the secretion of Th17-related cytokines (IL-17, IFNγ, IL-21, and IL-22) (36). These immunomodulatory effects directly disrupt the core autoimmune cascade underlying TAO, a T cell-mediated organ-specific autoimmune disease characterized by imbalances among T helper cell subsets (Th1/Th2/Th17/Treg) and excessive pro-inflammatory cytokine secretion (37, 38).

Second, VitD can directly modulate glucocorticoid sensitivity by regulating the expression and function of glucocorticoid receptor (GR). VitD supplementation elevates GR-α (but not GR-β) in severe asthma patients’ peripheral blood cells, improving treatment responses (39). This synergy extends to multiple sclerosis (MS), where VitD enhances GC efficacy by upregulating T-cell membrane glucocorticoid receptors (GR) (40), as evidenced by the fact that it cannot be replicated in GR-deficient mice (41). Nuclear colocalization of GR and VDR confirms GR as the pivotal mediator for VitD’s GC-potentiating effects (41). Consequently, these mechanistic insights explain the association between elevated VitD levels and enhanced therapeutic responses to IVGC observed in our cohort.

Δ25(OH)D exhibits enhanced predictive efficacy in specific subpopulations, particularly male patients, elderly patients (≥50 years), and those with pre-treatment 25(OH)D deficiency. Animal studies confirmed sex-specific heterogeneity in VitD’s effects on muscle tissue, with testosterone demonstrating synergistic potentiation of both muscular strength and molecular responses to VitD (42). Furthermore, multiple studies confirm marked age- and sex-dependent variations in VDR polymorphisms, which may relate to differential downstream functions and clinical outcomes mediated by the VitD/VDR pathway (43, 44). Additionally, research indicates that as age increases, VitD deficiency progressively worsens (45), and the expression of VitD 1α,25-hydroxylase decreases, ultimately leading to reduced levels of active VitD (46). Animal studies confirm that VitD deficiency reduces VDR expression, which subsequently elevates tumor necrosis factor-α (TNF-α) levels and exacerbates inflammation. Conversely, VitD supplementation and increase upregulate VDR, thereby suppressing TNF-α and mitigating inflammation (47).

This study offers new insights into VitD supplementation for TAO patients. While such supplementation remains controversial for TAO patients (a multicenter randomized double-blind trial in GD patients during ATD treatment showed no significant results (48), its necessity during IVGC therapy for TAO patients is clear (5). Traditionally recommended to prevent GC-induced osteoporosis (supported by post-treatment declines in osteoclast marker β-CTX in this study), VitD may also exert a GC therapy “sensitizing effect” independent of baseline levels—even marginal increases may boost IVGC efficacy. This highlights the need for continuous VitD monitoring in TAO patients during treatment, with enhanced supplementation for suboptimal responders or special populations (males, elderly, VitD -deficient individuals).

The study had some limitations. Firstly, this study is a retrospective cohort study, and the sample is limited. Compressive optic neuropathy is the most severe, sight-threatening manifestation of TAO that requires timely and standardized intervention; however, due to the limited sample size, we were unable to perform a stratified analysis of patients with this complication in the current study. Therefore, future studies are warranted to specifically explore the association between the presence of compressive optic neuropathy, Δ25(OH)D, and IVGC treatment response. Meanwhile, a large-scale prospective randomized controlled trial is warranted to further validate the conclusions of this study. Secondly, since all the TAO patients were recruited from northwest China, where VitD deficiency is highly prevalent and regionally heterogeneous, the study may have limited guiding significance for other populations. Thirdly, constrained by current detection methodologies, this study measured serum 25(OH)D rather than its bioactive terminal metabolite, 1,25(OH)_2_D. This limitation necessitates resolution through future technological advancements.

## 5 Conclusion

In conclusion, we demonstrated that Δ25(OH)D (change in serum 25(OH)D during therapy), rather than baseline 25(OH)D levels, emerged as an independent predictor of IVGC response in TAO patients, with enhanced predictive efficacy in male patients, elderly individuals (≥50 years), and those with pre-treatment 25(OH)D deficiency. Moreover, achieving a Δ25(OH)D threshold of +2.7 ng/mL was identified as a critical determinant for therapeutic success. This finding provides new insights into the immunomodulatory function of VitD in TAO pathogenesis, and underscores the necessity for continuous monitoring and optimized VitD repletion during IVGC therapy in high-risk subgroups.

## Data Availability

The raw data supporting the conclusions of this article will be made available by the authors, without undue reservation.
