# Supplementary material for: Vitamin D dynamics predict treatment response to intravenous glucocorticoids in thyroid-associated ophthalmopathy: a retrospective cohort study
Source: Front Immunol. 2026 Apr 21;17:1778702. doi: 10.3389/fimmu.2026.1778702 (PMC13139022; doi:10.3389/fimmu.2026.1778702)
Supplement: Supplementary file 1 [file Table1.doc]

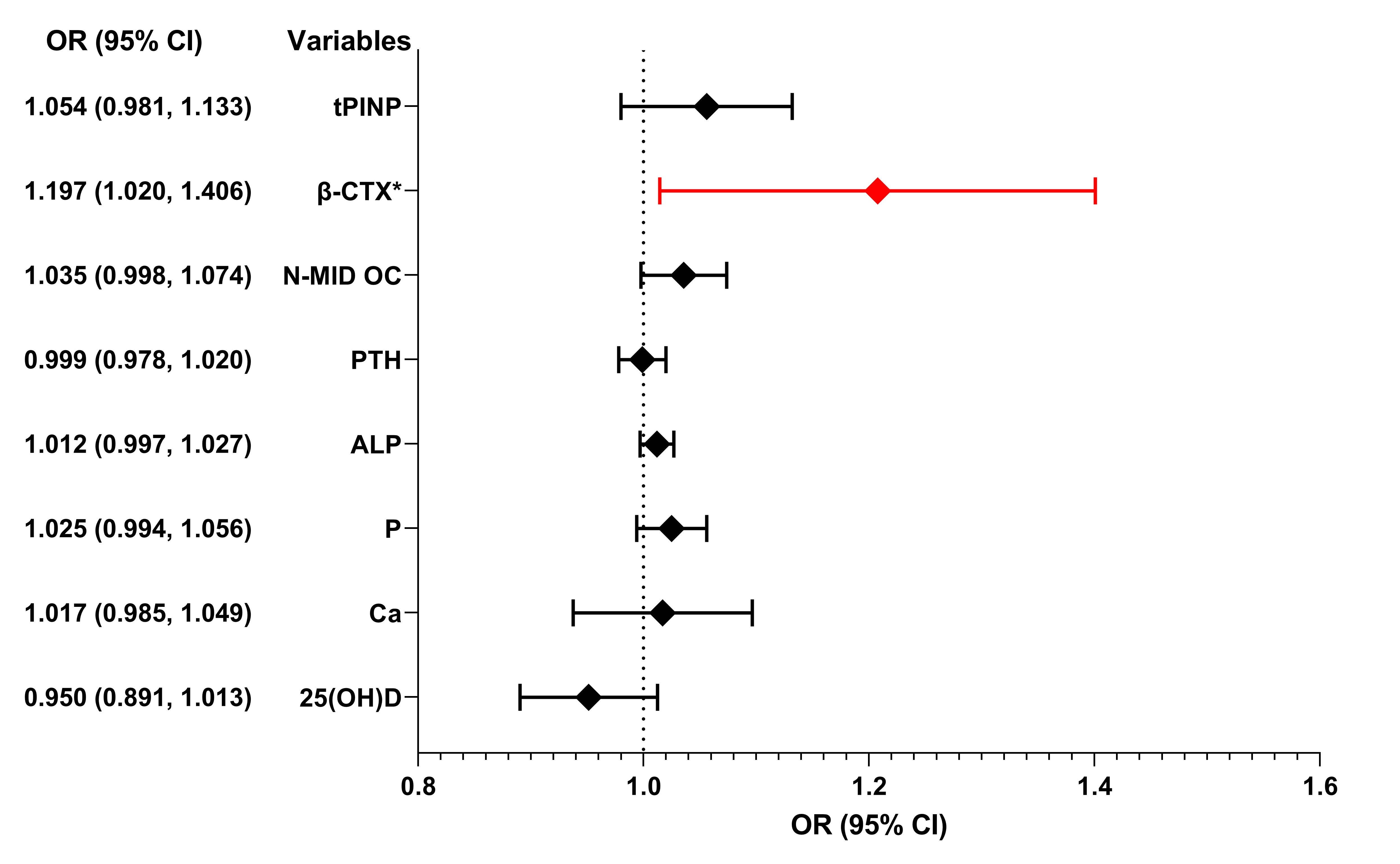


**Supplementary Figure 1. Forest graph showing the results of univariate logistic regression analyses of the association between baseline bone metabolism markers and the efficacy of IVGC.** OR, odds ratio; CI, confidence interval; tPINP, total procollagen type I N-terminal propeptide; β-CTX, β-CrossLaps; N-MID OC, N-terminal mid-fragment osteocalcin; PTH, parathyroid hormone; ALP, alkaline phosphatase; P, phosphorus; Ca, calcium; 25(OH)D, 25-hydroxyvitamin D. **P* < 0.05.
